# Guidelines for Optimisation of a Multiplex Oligonucleotide Ligation-PCR for Characterisation of Microbial Pathogens in a Microsphere Suspension Array

**DOI:** 10.1155/2015/790170

**Published:** 2015-02-03

**Authors:** Véronique Wuyts, Nancy H. C. Roosens, Sophie Bertrand, Kathleen Marchal, Sigrid C. J. De Keersmaecker

**Affiliations:** ^1^Department of Microbial and Molecular Systems, KU Leuven, Kasteelpark Arenberg 20, Bus 2460, 3001 Leuven, Belgium; ^2^Platform Biotechnology and Molecular Biology, Scientific Institute of Public Health (WIV-ISP), Juliette Wytsmanstraat 14, 1050 Brussels, Belgium; ^3^National Reference Centre for Salmonella and Shigella, Bacterial Diseases Division, Communicable and Infectious Diseases, Scientific Institute of Public Health (WIV-ISP), Juliette Wytsmanstraat 14, 1050 Brussels, Belgium; ^4^Department of Plant Biotechnology and Bioinformatics, Ghent University, Technologiepark 927, 9052 Ghent, Belgium; ^5^Department of Information Technology, Ghent University, IMinds, Gaston Crommenlaan 8, 9050 Ghent, Belgium

## Abstract

With multiplex oligonucleotide ligation-PCR (MOL-PCR) different molecular markers can be simultaneously analysed in a single assay and high levels of multiplexing can be achieved in high-throughput format. As such, MOL-PCR is a convenient solution for microbial detection and identification assays where many markers should be analysed, including for routine further characterisation of an identified microbial pathogenic isolate. For an assay aimed at routine use, optimisation in terms of differentiation between positive and negative results and of cost and effort is indispensable. As MOL-PCR includes a multiplex ligation step, followed by a singleplex PCR and analysis with microspheres on a Luminex device, several parameters are accessible for optimisation. Although MOL-PCR performance may be influenced by the markers used in the assay and the targeted bacterial species, evaluation of the method of DNA isolation, the probe concentration, the amount of microspheres, and the concentration of reporter dye is advisable in the development of any MOL-PCR assay. Therefore, we here describe our observations made during the optimisation of a 20-plex MOL-PCR assay for subtyping of* Salmonella * Typhimurium with the aim to provide a possible workflow as guidance for the development and optimisation of a MOL-PCR assay for the characterisation of other microbial pathogens.

## 1. Introduction

Characterisation of microbial pathogens beyond the species and subspecies level, that is, subtyping, requires more than one marker and these markers are usually combined in a multiplex assay for time-effectiveness of the assay. Evaluation of a multiplex assay can be facilitated by the Luminex technology, which has the capacity to analyse up to 500 markers in a single sample. Luminex assays are bead-based suspension arrays in which, in the case of DNA-based assays, fluorescently labelled oligonucleotides hybridise to probes that are coupled to distinctly coloured microspheres (up to 500 different colours). Fluorescently labelled oligonucleotides can be created by different types of assays, such as multiplex PCR (direct hybridisation assay), oligo ligation assay (OLA), allele-specific primer extension (ASPE) [1], and multiplex oligonucleotide ligation-PCR (MOL-PCR).

MOL-PCR was first described by Deshpande et al. [2] as a powerful tool for detection of microbial pathogens allowing to combine analysis of multiple types of markers like unique sequences, indels, repeats, or single nucleotide polymorphisms (SNPs) in a single multiplex reaction. With MOL-PCR high levels of multiplexing can be achieved, because the multiplexing step is a ligation rather than a PCR and signals are amplified in a singleplex PCR. MOL-PCR is a variant on multiplex ligation-dependent probe amplification (MLPA) [3] in which the overnight hybridisation step and subsequent ligation are replaced by cycles of hybridisation and ligation by a thermostable ligase. The read-out of MLPA products occurs through fragment sizing by capillary electrophoresis [3], but also applications with analysis on a Luminex device have been reported [4, 5].

Multiplex ligation-based assays as MOL-PCR and MLPA have been reported as efficient assays for the diagnosis of human genetic diseases [3, 6–10], the detection of viruses [11–13] and bacteria [2, 14, 15], and characterisation of pathogens, including subtyping [16–21].

Although MOL-PCR is increasingly used for characterisation of microbial pathogens on pure isolates, little is found in literature on the steps taken during optimisation of the assay, leading to the final, published protocol. A paper on* Mycobacterium tuberculosis* refers to a general protocol and gives little detail on the reaction conditions [5]. Deshpande et al. [2], Stucki et al. [18] and Thierry et al. [20] provide the reaction conditions in detail, but refrain from comprehensive optimisation results, although encountered issues with some aspects in the assay and their solutions are discussed by Thierry et al. [20].

As we consider that optimisation experiments might contain valuable information for other scientists starting with the development of a MOL-PCR assay for their microbial pathogen of interest, we describe here the observations we made during the optimisation process of a 20-plex MOL-PCR assay, which is one of three MOL-PCR assays for the subtyping of* Salmonella enterica *subsp.* enterica* serovar Typhimurium (*S*. Typhimurium), as an illustration of which parameters could be optimised for a MOL-PCR assay for other pathogens.

Even though optimisation of MOL-PCR might depend on the markers in the assay and on the bacterial species for which the assay is intended, we point out the main parameters of the MOL-PCR assay that could be evaluated during the design of a new assay for another microbial pathogen.

## 2. Materials and Methods

### 2.1. Probe Design

The MOL-PCR assay taken as an example in this study contains 18 discriminating markers and 2 markers as internal positive control of the DNA template. One internal positive control marker targets a SNP, while all other markers detect the presence of a unique sequence. Each marker requires an upstream and downstream probe that anneal adjacent to each other on the target DNA. The upstream probes consist of a 5′ 20 bp T7 primer sequence, an internal 24 bp anti-TAG sequence, and a 3′ 19 bp to 27 bp target-specific sequence. The downstream probes are made up of a 5′ 18 bp to 28 bp target-specific sequence and at 3′ the 20 bp reverse-complement of a T3 primer. The downstream probes are 5′ phosphorylated to enable ligation to the upstream probe by the DNA ligase. All probe pairs were designed with VisualOMP (DNA Software) so that the complex of target-specific DNA, upstream probe, and downstream probe had an effective change of Gibb's free energy (Eff ΔG°) of −31.15 to −25.13 kcal mol^−1^ and a melting temperature of 56 to 64°C at the conditions of the multiplex ligation reaction, except for one discriminating and one internal DNA control marker for which the complex had a melting temperature of 68°C and 52°C, respectively. As the aim of this study is not to provide a new subtyping method for* S.* Typhimurium, but to elaborate on important parameters to be evaluated during the development of a MOL-PCR assay, the sequences of the probes and primers used are less relevant for the key message of this paper. However, sequences of probes (partially based on Fang et al. [22]) and primers are available upon request. All probes and primers were ordered from Eurogentec with a RP-Cartridge-Gold purification for upstream probes and T7 primer and a reverse phase HPLC purification for downstream probes and biotinylated T3 primer. A HPLC purification was chosen for the downstream probes and T3 primer, since the supplier did not offer the basic RP-Cartridge-Gold purification for these modified oligonucleotides.

### 2.2. Bacterial Isolates and DNA Isolation

All *S*. Typhimurium isolates in this study were received from the Belgian National Reference Centre for* Salmonella *and* Shigella*. The test panel consisted of 6* S*. Typhimurium isolates, which were selected so that for each of the 18 discriminating markers at least 1 positive and 1 negative result was obtained.

Different methods for DNA isolation based on heat lysis were tested, that is, heat lysis in water and heat lysis in a commercial product for DNA purification were adapted for a single colony from previous reports [5, 18, 20, 23] (Table 1: DNA isolation). Isolates were grown overnight (14 to 20 hours) at 37°C on LB agar (Merck). In the first method, a single colony was dissolved in 50 to 300 *μ*L sterile deionised water and incubated at 100°C for 10 min. After cooling for at least 5 min at 4°C and centrifugation for 10 min at 11000 g, the supernatant was stored at −20°C and used for further analysis. In the second method, a single colony was treated as described in the product insert of the xMAP* Salmonella* Serotyping Assay Kit [23]. In short, a single colony was added to 20 *μ*L InstaGene Matrix (Bio-Rad) and in a thermal cycler the following programme was run: 56°C for 10 min, 99°C for 5 min, and 4°C forever. After addition of 100 *μ*L of nuclease free distilled water (Life Technologies), the tube was spun for 5 min in a microcentrifuge and 50 *μ*L of the supernatant was stored at −20°C for further use.

A positive control template DNA was created by combining a single colony of each of 5 different isolates in one tube, which was treated in the same way as the isolates of the test panel. The combination of these 5 isolates yielded a positive reaction for each of the 20 markers in the assay.

### 2.3. Multiplex Oligonucleotide Ligation

The multiplex ligation reaction mix combined 1 to 5 nM of each of the 40 probes with 1X* Taq *DNA ligase reaction buffer (New England BioLabs), 2 to 6 units of* Taq *DNA ligase (New England BioLabs), and 2 or 4 *μ*L of template DNA and was brought to a final volume of 10 *μ*L with nuclease free distilled water (Life Technologies). The thermal cycling programme (Swift MaxPro, Esco) included 5 or 10 min of denaturation at 95°C followed by 30 cycles of 25 s at 94°C and 30 s at 58°C (Table 1: Multiplex oligonucleotide ligation). Each experiment included a positive control for the reaction and a no-template-control (NTC) to measure background signal for which the template DNA was replaced by, respectively, positive control template DNA and nuclease free distilled water. The results of neither the positive nor the negative control were taken into account for the statistical analysis and are not included in the figures shown, since these results are not representative of a bacterial isolate to be characterised.

### 2.4. Singleplex Polymerase Chain Reaction

The singleplex PCR reaction (Table 1: PCR) was performed in a final volume of 10 *μ*L which included 1X HotStarTaq PCR buffer (Qiagen), 125 nM T7 primer (Eurogentec), 500 nM 5′-biotin-T3 or 5′-Alexa 532-T3 primer (Eurogentec), 200 *μ*M of each dNTP (Thermo Scientific), 0.25 or 0.5 units HotStarTaq DNA polymerase (Qiagen), and 3 or 5 *μ*L of ligase product. The following protocol was run in a thermal cycler (Swift MaxPro, Esco): 15 min at 95°C, 35 to 40 cycles of 30 s at 94°C, 30 s at 60°C, and 30 s at 72°C, 5 min at 72°C.

### 2.5. Hybridisation to Microspheres and Analysis on Luminex Platform

An adapted version of the manufacturer's no-wash protocol [1] was used for the hybridisation reaction (Table 1: Hybridisation and analysis on MAGPIX). Twenty regions of MagPlex-TAG microspheres (Luminex) with anti-TAGs specific to each of the probe pairs in the assay were diluted/concentrated to 350 to 2500 microspheres of each region per reaction in Tm hybridisation buffer. In a total volume of 25 *μ*L, 2.5 *μ*L, 5 *μ*L, or all of the PCR product was combined with the microsphere mix to a final concentration of 1X Tm hybridisation buffer (0.1 M Tris-HCl pH 8.0 (Sigma), 0.2 M NaCl (Sigma), and 0.08% Triton X-100 (Sigma) in nuclease free distilled water (Life Technologies)). All of the PCR product is defined as the complete content of the tube after the PCR, which may be less than the initial 10 *μ*L volume attributable to possible evaporation. In a thermal cycler (Swift MaxPro, Esco; or iCycler, Bio-Rad), the samples were denatured for 90 s at 96°C and hybridisation to anti-TAGs on the microspheres occurred for 30 min at 37°C. In case a 5′-biotin-T3 primer was used in the PCR, 100 *μ*L of a reporter mix including 4 or 10 *μ*g/mL of streptavidin-R-phycoerythrin (SAPE) (Life Technologies) in 1X Tm hybridisation buffer was added to each sample and after incubation for 15 min at 37°C in a thermal cycler (Swift MaxPro, Esco; or iCycler, Bio-Rad), 100 *μ*L of the sample was analysed on a MAGPIX device (Luminex). In case a 5′-Alexa 532-T3 primer was used in the PCR, 100 *μ*L of 1X Tm hybridisation buffer was added to each sample before analysis of 100 *μ*L on a MAGPIX device.

The analysis was performed at 37°C. The protocol included a sample wash in the MAGPIX device and the minimum bead count was 50 microspheres of each region.

### 2.6. Statistical Analysis

The output of the MAGPIX device includes the median fluorescence intensity (MFI) value for each marker, that is, for each DNA sample or control 20 MFI values are obtained, since the MOL-PCR is a 20-plex assay. These MFI values were read into R software [24]. Signal-to-noise ratios (SN) were calculated by dividing the MFI of the sample by the corresponding MFI of the NTC (1). As such, each sample yielded 20 signal-to-noise ratios:

(1) $$\begin{array}{c} {\text{SN}}_{{\text{sample}}_{\text{marker }a}}=\frac{{\text{MFI}}_{{\text{sample}}_{\text{marker }a}}}{{\text{MFI}}_{\text{NT}{\text{C}}_{\text{marker }\;a}}}. \end{array}$$

In each experiment, different test conditions were compared to a reference condition. For each condition, the test panel of 6* S*. Typhimurium isolates generated 6 × 20 = 120 signal-to-noise ratios, of which 56 signal-to-noise ratios were expected to be positive and 64 were expected to be negative based on a prior screening with PCR or sequencing. The 56 positive signal-to-noise ratios of each test condition were compared to the 56 positive signal-to-noise ratios of the reference condition using a paired Wilcoxon signed-rank test, for which the two-sided variant tests that the distribution of the difference of signal-to-noise ratios of reference and test condition comes from a distribution symmetric around 0. This nonparametric alternative for the *t*-test was used since, with Kolmogorov-Smirnov tests on each set of 56 positive signal-to-noise ratios, the null hypothesis of normal distribution had to be rejected at significance level 0.01.

Each experiment was performed twice in an independent manner and the results of each test were statistically analysed separately. For each experiment, the results of only one of the 2 independent tests are presented, since each of the 2 independent tests yielded the same statistical results. The results of the positive control for the reaction (using the positive control DNA template) and of the NTC (nuclease free distilled water as template) are not included in the figures. For example, in the first experiment the test conditions are 3 different methods for DNA isolation and those are compared to a previously used method for DNA isolation, which is the reference condition. As the panel of 6* S*. Typhimurium isolates together with the positive control for the reaction and a NTC were assayed twice for each of the 4 DNA isolation methods, the first experiment included 8 (6 isolates, 1 positive control, 1 NTC) × 4 (conditions) × 2 (independent repetitions) = 64 MOL-PCR assays, consisting of 1280 signal-to-noise ratios in total (20 × (8 × 4 × 2)). However, only the results of the test panel (6 isolates) in the 4 conditions and of one of 2 independent tests are shown, so that in Figure 2 the results of 6 × 4 = 24 MOL-PCR assays (24 × 20 = 480 signal-to-noise ratios) are presented, representative of the 2 independent repetitions. The significance level was set at 0.01 for all hypothesis tests. All statistical analyses were performed in R software [24].

## 3. Results and Discussion

**Figure 1:**
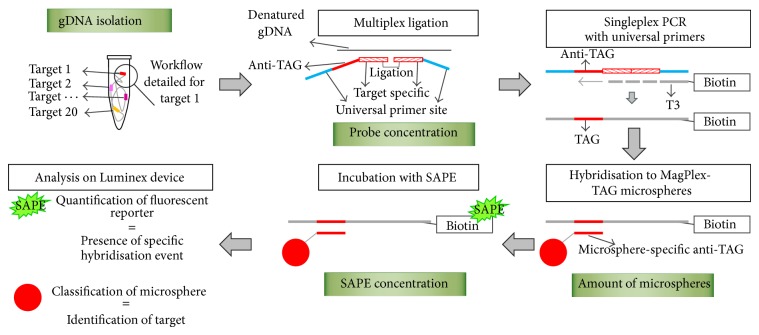
Overview of a multiplex oligonucleotide ligation-PCR (MOL-PCR) assay. The workflow is illustrated for 1 target, with the described MOL-PCR being a 20-plex assay. However, a MAGPIX device can discriminate up to 50 different microsphere sets and hence up to 50 different targets. Optimisation of the MOL-PCR assay was attempted for several parameters and the green boxes indicate those parameters which had a major influence on the signal-to-noise ratios, as has been elaborated in the text. gDNA: genomic DNA; SAPE: streptavidin-R-phycoerythrin; see text for more details.

MOL-PCR with separated ligation and PCR [18, 20] includes 3 main steps (see Figure 1 for an overview of the assay). The first step after DNA isolation is a multiplex oligonucleotide ligation in which a pair of probes that anneal adjacent to each other on the target DNA sequence is ligated by a thermostable DNA ligase. For each marker in the multiplex assay, a different probe pair is included in the assay. Ligation of probe pairs results in various single-stranded DNA molecules, which function as a template in the subsequent singleplex PCR with a universal primer pair (e.g., T3 and T7). One primer is biotinylated for read-out on a Luminex device. In the third step, the PCR products are hybridised to MagPlex-TAG microspheres, each with a different colour shade of red, through a TAG that is incorporated in the marker-specific probes and that is complementary to the anti-TAG coupled to the microspheres. For each marker a different TAG and a different MagPlex-TAG microsphere is used in the assay. After incubation with streptavidin-R-phycoerythrin (SAPE), a Luminex device will identify the microsphere based on the measurement of the red colour, and thus the marker, and measure the fluorescence signal of the SAPE to detect whether a PCR product has hybridised to the anti-TAG on the microsphere.

The optimisation pursues high signal-to-background ratios for positive results, which can be achieved with low background fluorescence intensity and high fluorescence intensity for positive results. As this MOL-PCR assay is intended for use as a routine subtyping method, cost is also an important factor in the optimisation.

Optimisation of a MOL-PCR assay can be attempted at different parameters of the preparatory work and in each of the 3 steps of the assay (Figure 1). Here, we used a MOL-PCR assay in development as a routine subtyping method for *S*. Typhimurium to illustrate which parameters are important to be evaluated and, if needed, to be adapted, to achieve high signal-to-noise ratios. Different conditions were tested to check whether deviations from the standard values (based on literature and Luminex guidelines) had a significant impact on the signal-to-noise ratios and would thus be advisable for further evaluation during optimisation of a MOL-PCR assay. If no impact was seen, no further conditions were tested. The optimal conditions for the parameter that was tested in one experiment were further used in the following experiments. Table 1 summarises all tested parameters, conditions, and the results of the statistical analysis, which are elaborated below. Also issues that emerged during this evaluation of the assay are discussed below.

### 3.1. Probe Premixes

Since the described MOL-PCR assay is intended for routine use, the preparation of a complex ligation mix with addition of each of the 40 probes separately each time the assay is performed, should be avoided. Therefore, it was attempted first to combine different probe pairs in a defined number of different probe premixes. To avoid repetitive freeze-thaw cycles of diluted oligonucleotides, these probe premixes were stored at −20°C in single-use aliquots at a concentration of 5 *μ*M. However, some combinations of probes in one premix resulted in high background signals as measured by the MFI in the NTC (data not shown). These high background signals were eliminated by changing probe pairs from one probe premix to another or by taking the probe pair out of the probe premixes. Finally, the 40 probes were divided over 3 probe premixes including 8, 5, and 3 probe pairs each and the last 8 probes were separately added to the ligation mix. The latter 8 probes were also stored in a concentration of 5 *μ*M at −20°C in single-use aliquots. It was observed that a combination of these 8 probes in a probe premix that was not frozen before use did not result in high background signals. This clearly illustrates that the storage and combination of probes may have an impact on the background signals and therefore on the signal-to-noise ratios.

### 3.2. DNA Isolation

A simple, time- and cost-efficient method for DNA isolation is preferable for routine purposes. Heat lysis of bacterial cultures meets these requirements and was previously described in literature [5, 18, 20] for preparation of a DNA template for a MOL-PCR assay. Therefore, in the first experiment, different methods for DNA isolation by heat lysis were compared. The reference condition was heat lysis of a single colony in 300 *μ*L sterile deionised water, which was previously used for PCR screening of the markers. Test conditions with more concentrated DNA template were heat lysis of a single colony in 100 *μ*L or 50 *μ*L sterile deionised water or in 20 *μ*L of InstaGene Matrix, which is a commercial kit. By using DNA of a single colony, potential inaccurate characterisation results assignable to a possible mixture of different strains can be avoided, which is not the case when DNA is isolated from, for example, liquid cultures or multiple colonies. Other parameters for this MOL-PCR assay are shown in Table 1 as experiment 1.

**Figure 2:**
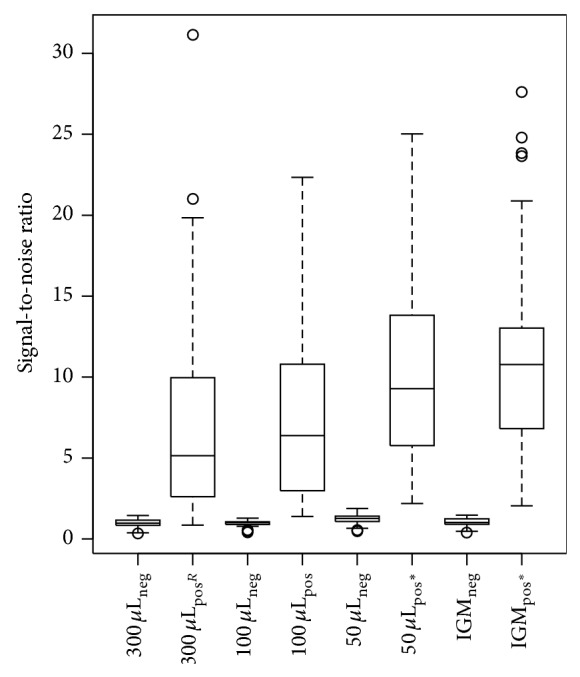
Boxplot of signal-to-noise ratios for different methods of DNA isolation. Results of one of 2 independent tests are presented, that is, results of 6 isolates × 4 conditions = 24 MOL-PCR assays (for each of the 4 conditions: *n*_total_ = 120 with *n*_neg_ = 64 and *n*_pos_ = 56). 300 *µ*L: single colony in 300 *μ*L sterile deionised water; 100 *µ*L: single colony in 100 *μ*L sterile deionised water; 50 *µ*L: single colony in 50 *μ*L sterile deionised water; IGM: single colony in 20 *μ*L InstaGene Matrix; neg: expected negative results; pos: expected positive results; R: reference condition; ^*^: statistically significant difference with the reference condition.

A boxplot of the signal-to-noise ratios for the different methods of DNA isolation is given in Figure 2. The two-sided hypothesis test did not demonstrate a significant difference between the positive signal-to-noise ratios of a single colony in 300 *μ*L and 100 *μ*L sterile deionised water. *P* values of one-sided hypothesis tests between reference and a single colony in 50 *μ*L sterile deionised water and between reference and a single colony in 20 *μ*L InstaGene Matrix indicated significant improvements by the test conditions. This significant difference can be explained by the higher MFI values that were obtained for the test panel with DNA templates of heat lysis in 50 *μ*L water and in InstaGene Matrix in comparison to the reference condition (Data set S.1). More concentrated DNA is thus beneficial for the MOL-PCR assay.

The boxplot shown in Figure 2 indicates that heat lysis in InstaGene Matrix results in higher signal-to-noise ratios than heat lysis in 50 *μ*L deionised water. However, heat lysis of a single colony in 50 *μ*L sterile deionised water is preferred over heat lysis of a single colony in InstaGene Matrix, since heat lysis in water is cheaper than heat lysis in InstaGene Matrix, which makes heat lysis in water more convenient for DNA isolation in a routine method for bacterial pathogen characterisation.

### 3.3. Multiplex Oligonucleotide Ligation

Next, the impact of the ligation mix with the concentration of the probes, the added volume of DNA template and the quantity of* Taq* DNA ligase enzyme, and of the time of initial denaturation of the DNA during the multiplex oligonucleotide ligation was evaluated.

In the literature, the concentration of the probes ranges from 0.25 nM [5] up to 10 *μ*M [20]. To evaluate the effect of the probe concentration on the signal-to-noise ratio, the concentration of each of the 40 probes in the ligation mix was tested at 1 nM and 5 nM [2] with 2 nM [18] as reference condition. Other parameters for this MOL-PCR assay are shown in Table 1 as experiment 2.

**Figure 3:**
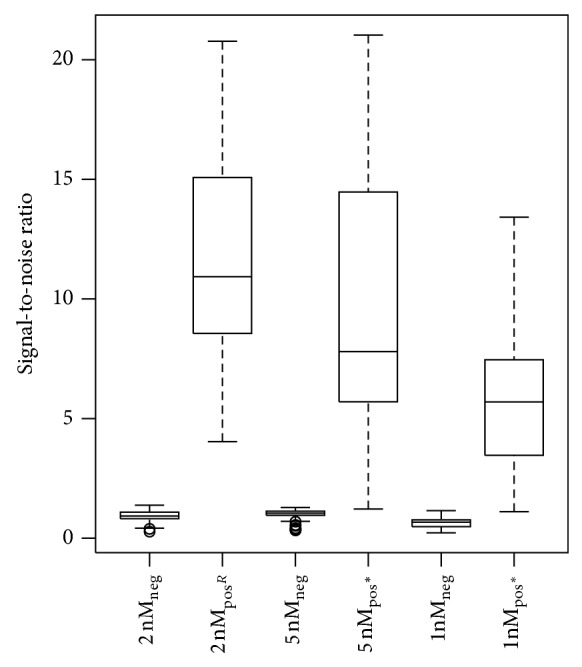
Boxplot of signal-to-noise ratios for different probe concentrations in the ligation mix. Results of one of 2 independent tests are presented, that is, results of 6 isolates × 3 conditions = 18 MOL-PCR assays (for each of the 3 conditions: *n*_total_ = 120 with *n*_neg_ = 64 and *n*_pos_ = 56). neg: expected negative results; pos: expected positive results; R: reference condition; ^*^: statistically significant difference with the reference condition.

A boxplot of the signal-to-noise ratios for the different probe concentrations is given in Figure 3. For both 1 nM and 5 nM probe concentrations, the one-sided hypothesis tests demonstrated significant differences in favour of the reference condition. Compared to the reference condition of 2 nM probe concentration, higher MFI values were seen for the NTC at 1 nM probe concentration (Data set S.2) and lower MFI values for expected positive results in the test panel at 5 nM probe concentration (Data set S.3). The total amount of probes in a MOL-PCR reaction seems to be a crucial factor. Therefore, the optimal individual probe concentration might be dependent on the level of multiplex of the MOL-PCR assay, as remarked in literature [5, 18] and based on our own experience. Indeed, the MLPA assay of Bergval et al. [5] included 47 informative probe pairs (high multiplex level) and hybridisation occurred at a concentration of 0.25 nM of each probe, whereas a concentration of 5 nM of each probe gave high signal-to-noise ratios for positive results in our initial MOL-PCR tests with 6 probe pairs (low multiplex level) (data not shown). At an intermediate level of multiplex, the MOL-PCR assay of Stucki et al. [18] which interrogated 8 SNPs with 3 probes per SNP used a protocol with an intermediate concentration of 2 nM of each probe.

As the cost of a routine assay should be kept as low as possible, the number of* Taq *DNA ligase units is an important aspect for optimisation, since enzymes are a key factor in the cost of a reaction. In experiment 3, the combination of the number of ligase enzyme units and the volume of DNA template in the ligation mix was evaluated, since a possible higher yield of ligated probe pairs due to a higher concentration of enzyme might be restricted by the availability of DNA template. As reference condition the combination of 2 *μ*L DNA template with 2 units of ligase enzyme, the least expensive, was taken, whereas test conditions were all 5 other combinations of 2 or 4 *μ*L DNA template [18, 20] with 2, 4 [18], or 6 units of ligase enzyme (see Table 1 for other parameters of this MOL-PCR assay).

A boxplot of the signal-to-noise ratios for the different conditions is presented in supplementary Figure S.1 in supplementary material available online at http://dx.doi.org/10.1155/2015/790170. One-sided hypothesis tests indicated significant differences in support of the reference condition for all test conditions. With increasing units of ligase enzyme in combination with 2 *μ*L of DNA template, increasing MFI values for the NTC were seen (Data set S.4), which caused declining signal-to-noise ratios. At 2 units of ligase enzyme, MFI values for the test panel were generally lower for 4 *μ*L of DNA template than for 2 *μ*L of DNA template (Data set S.5), which might be explained by inhibition of the ligation reaction because of impurities in the DNA extract. Therefore, pure DNA extracted from liquid cultures with a commercial kit might ameliorate the ligation reaction when more template DNA is used. However, a trade-off should be made between the advantage of using pure DNA and the associated disadvantages of cost, time, and possible strain variations occurred during culturing.

In a fourth experiment, the initial denaturation of the DNA template was considered. An initial denaturation of 10 min at 95°C was previously used in the PCR for screening of the markers. However, as a longer time at an elevated temperature may have a negative effect on the activity of the ligase enzyme, a shorter initial denaturation time was tested. An initial denaturation of 5 min and 10 min at 95°C served as reference and test condition, respectively (see Table 1 for other parameters of this MOL-PCR assay).

A boxplot of the signal-to-noise ratios for the different initial denaturation times is shown in supplementary Figure S.2. A significant difference in favour of the longer initial denaturation time is demonstrated by a one-sided hypothesis test. This can be explained by slightly higher MFI values for the NTC at 5 min initial denaturation, while the MFI values for the test panel stays at the same level with both denaturation times (Data set S.6). As such, a potential higher activity of the ligase enzyme due to a shorter time of denaturation at an elevated temperature does not produce high enough MFI values for the test panel to counteract the increased MFI values of the NTC (Data set S.6).

### 3.4. Singleplex Polymerase Chain Reaction

Subsequently, the singleplex PCR was assessed at the volume of ligation product added, the number of DNA polymerase units used, and the number of PCR cycles performed.

In experiment 5, addition of 3 *μ*L [18] or 5 *μ*L (half of the total volume) of ligation product to the PCR mix was compared (see Table 1 for other parameters of this MOL-PCR assay) and supplementary Figure S.3 shows the resulting signal-to-noise ratios. A two-sided hypothesis test did not provide sufficient evidence for a significant difference between both volumes of ligation product. Yet, we selected 3 *μ*L of ligation product as optimal condition, because the ligation reaction volume is 10 *μ*L and after taking 3 *μ*L of the ligation product there would still be enough ligation product left to repeat the PCR if necessary.

As already indicated in the multiplex oligonucleotide ligation reaction, enzymes are an important cost in an assay. However, a balance should be sought between cost and efficacy of the reaction. Therefore, 0.25 units and 0.5 units of HotStarTaq DNA polymerase were evaluated in experiment 6 (see Table 1 for other parameters of this MOL-PCR assay), for which the resulting signal-to-noise ratios are given in supplementary Figure S.4. A one-sided hypothesis test pointed out a significant difference in support of 0.25 units of DNA polymerase. Indeed, the MFI values for the NTC with 0.5 units of DNA polymerase were elevated such that, although higher MFI values for the test panel were obtained, the signal-to-noise ratios were considerably lower than those of the PCR with 0.25 units DNA polymerase (Data set S.7).

These results are in line with the recommendations of Qiagen [25] to use 2.5 units of HotStarTaq DNA polymerase in a PCR reaction with a total volume of 100 *μ*L, which corresponds to 0.25 units of DNA polymerase in a 10 *μ*L PCR reaction.

A higher number of cycles in the PCR reaction may produce higher MFI values for positive results but also increases the turnaround time of the assay. The number of PCR cycles ranges in the literature from 35 [5] up to 45 [2, 20] and Luminex [1] suggests 35 cycles for PCR. To evaluate if a higher number of PCR cycles had a positive influence on the signal-to-noise ratios, 40 cycles were tested. Supplementary Figure S.5 illustrates the signal-to-noise ratios for 35 and 40 PCR cycles (see Table 1 for other parameters of this MOL-PCR assay). A significant difference in favour of 35 PCR cycles was demonstrated by a one-sided hypothesis test. Compared to the PCR with 35 cycles, the PCR with 40 cycles resulted in higher MFI values for the test panel, but they did not lead to increased signal-to-noise ratios caused by raised MFI values for the NTC (Data set S.8).

### 3.5. Thermal Cycler Used for the MOL-PCR Assay

During the evaluation experiments, it was observed that the thermal cycler had an influence on the MFI values for the NTC (data not shown). When the multiplex oligonucleotide ligation or the PCR or both reactions were performed on an iCycler (Bio-Rad), higher MFI values for the NTC were obtained than for MOL-PCR assays completely run on a Swift MaxPro (Esco) thermal cycler. An apparent difference between both thermal cyclers is the maximum heating and cooling rate, which is 3.3°C/s and 2.0°C/s for, respectively, heating and cooling in the iCycler, and 4°C/s for both heating and cooling in the Swift MaxPro thermal cycler. A lower ramp rate might thus enhance cross-reactivity of the probes, which is measured through the NTC. This parameter should be checked before starting the MOL-PCR assay development.

### 3.6. Hybridisation to Microspheres and Analysis on Luminex Platform

For optimisation of the hybridisation of the PCR product to the MagPlex-TAG microspheres and read-out on a MAGPIX device, the volume of PCR product added, the amount of microspheres, concentration of SAPE in the reporter mix, and the type of reporter dye were taken into account.

The manufacturer's no-wash protocol for hybridisation to MagPlex-TAG microspheres [1] recommends addition of 2.5 *μ*L of PCR product to the microsphere mix for the hybridisation reaction, while in the literature 5 *μ*L [20] to 10 *μ*L [2, 5, 18] is reported. A comparison was made with 2.5 *μ*L of PCR product as reference condition and 5 *μ*L of PCR product and all (theoretically 10 *μ*L) of the PCR product as test conditions (see Table 1 for other parameters of this MOL-PCR assay). The signal-to-noise ratios of this experiment are shown in supplementary Figure S.6. Both test conditions presented a significant difference against the reference condition as demonstrated by one-sided hypothesis tests.

In view of an assay for routine use, addition of 5 *μ*L of PCR product to the hybridisation reaction is preferred over addition of all PCR product, since standardisation of the assay is more straightforward with well-defined volumes.

The main costs in a MOL-PCR assay are the microspheres and SAPE as a fluorescent reporter. In the literature, 312.5 [18] up to 5000 [2] microspheres of each region are used per reaction. In experiment 9, an amount of 2500 microspheres of each region per reaction in combination with a concentration of 10 *μ*g/mL of SAPE in the reporter mix was taken as reference condition, as suggested in the Luminex no-wash protocol [1]. The test conditions were all 5 other combinations of 2500, 375, or 750 microspheres of each region per reaction in combination with a concentration of 4 or 10 *μ*g/mL SAPE in the reporter mix (see Table 1 for other parameters of this MOL-PCR assay).

**Figure 4:**
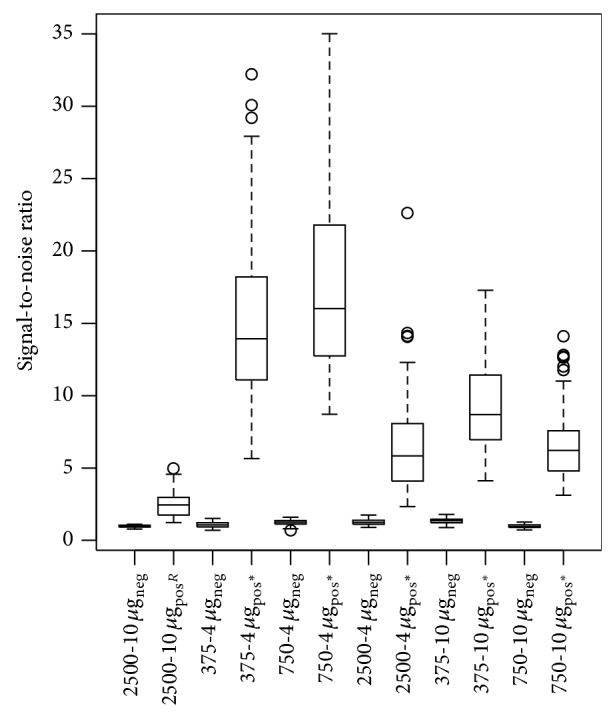
Boxplot of signal-to-noise ratios for different combinations of amount of microspheres in the hybridisation reaction and streptavidin-R-phycoerythrin (SAPE) concentration in the reporter mix. Results of one of 2 independent tests are presented, that is, results of 6 isolates × 6 conditions = 36 MOL-PCR assays (for each of the 6 conditions: *n*_total_ = 120 with *n*_neg_ = 64 and *n*_pos_ = 56). 2500: 2500 microspheres of each region per reaction were combined in the microsphere mix; 375: 375 microspheres of each region per reaction were combined in the microsphere mix; 750: 750 microspheres of each region per reaction were combined in the microsphere mix; 4 *µ*g: the reporter mix contained 4 *μ*g/mL of SAPE; 10 *µ*g: the reporter mix contained 10 *μ*g/mL of SAPE; neg: expected negative results; pos: expected positive results; R: reference condition; ^*^: statistically significant difference with the reference condition.

Figure 4 illustrates the signal-to-noise ratios of this experiment. One-sided hypothesis tests indicated significant improvements of all test conditions compared to the reference condition. Indeed, MFI values for the test panel were lower for an amount of 2500 microspheres in combination with both 4 and 10 *μ*g/mL SAPE in the reporter mix than for all other combinations (Data set S.9). In addition, a SAPE concentration of 10 *μ*g/mL in the reporter mix resulted in higher MFI values for the NTC at all 3 amounts of microspheres (Data set S.10). These findings lead to the conclusion that the reference condition generates the lowest signal-to-noise ratios for expected positive results.

At a SAPE concentration of 4 *μ*g/mL in the reporter mix, an amount of 750 microspheres of each region per reaction is preferred over 375 microspheres of each region per reaction, because microspheres counts below 50, which is the minimum bead count recommended by Luminex, were observed during analysis of reactions with 375 microspheres of each region.

A last experiment compared the use of SAPE and Alexa 532 as fluorescent reporter for the read-out on a MAGPIX device (see Table 1 for other parameters of this MOL-PCR assay). SAPE is recommended by Luminex as most suitable fluorescent reporter (100% relative fluorescence intensity) and Alexa 532 (28%) as second and Cy3 (19%) as third most suitable fluorescent reporter [26]. The use of a T3 primer coupled with Alexa 532 [2, 18] or Cy3 [5] has the advantage that an incubation with reporter mix is not needed, in contrast to a T3 primer coupled with biotin which binds to SAPE during the incubation with reporter mix.

The resulting signal-to-noise ratios of experiment 10 are given in supplementary Figure S.7. A one-sided hypothesis test demonstrated a significant difference in support of SAPE as fluorescent reporter. MFI values for both test panel and NTC are considerably lower with Alexa 532 as fluorescent reporter compared to SAPE, with decreased signal-to-noise ratios as a consequence with Alexa 532. As Alexa 532 did not perform better than SAPE and given that the relative fluorescence intensity of Cy3 is even lower than that of Alexa 532, Cy3 was not further tested.

## 4. Conclusion

By systematically optimising different parameters at each step of the assay, we have improved a MOL-PCR assay from an assay with no clear difference between positive and negative results (Figure 2) to an assay of which the signal-to-noise ratios present a clear difference between positive and negative results (Figure 4). A summary of the effect of each of the optimised parameters on the signal-to-noise ratios and on the cost of the assay is presented in Table 2. Evidently, the parameters which have a major effect on signal-to-noise ratios and/or on cost should be prioritised in an optimisation procedure during the development of a similar assay. Major improvements were obtained with changing the method of DNA isolation, lowering the probe concentration in the multiplex ligation mix, and reducing the amount of microspheres of each set per reaction and the concentration of SAPE in the reporter mix (Figure 1). We also found that the combination of probe pairs in a frozen premix and the type of thermal cycler used for the multiplex ligation and the singleplex PCR have an influence on the background signals measured through the NTC. These observations are taken into account during the currently ongoing development and validation of a full MOL-PCR assay for the subtyping of* S.* Typhimurium (Wuyts et al., in preparation). However, evaluation of the parameters for which a significant impact on the signal-to-noise ratios was seen, is worthwhile for characterisation of any microbial pathogen if the cost and effort of a MOL-PCR assay are important.

## Supplementary Material

The supplemental figures show the boxplots of signal-to-noise ratios of additional parameters. The supplemental data sets include the MFI values that were used to calculate the signal-to-noise ratios as background information to explain some of the results.

## Figures and Tables

**Table 1:** Test and reference conditions for the optimisation experiments.

Condition	Preparatory work				Multiplex oligonucleotide ligation										PCR						Hybridisation and analysis on MAGPIX									
	DNA isolation^a^				Probe concentration (nM)			DNA volume (*µ*L)		*Taq* DNA ligase (units)			Initial denaturation (min)		Ligation product volume (*µ*L)		*Taq* DNA polymerase (units)		Cycles		PCR product volume (*µ*L)			Microspheres per reaction			SAPE concentration (*µ*g/mL)		Reporter dye	
Experiment	300	100	50	IGM	1	2	5	2	4	2	4	6	5	10	3	5	0.25	0.5	35	40	2.5	5	all	350	750	2500	4	10	SAPE	Alexa 532

1	R	ns	**ls**	ls			x	x			x			x	x		x		x			x			x		x		x	
2			x		gs	**R**	gs	x			x			x	x		x		x			x			x		x		x	
3			x			x		**R**	gs	**R**	gs	gs		x	x		x		x			x			x		x		x	
4			x			x		x		x			R	**ls**	x		x		x			x			x		x		x	
5			x			x		x		x				x	**R**	ns	x		x			x			x		x		x	
6			x			x		x		x				x	x		**R**	gs	x			x			x		x		x	
7			x			x		x		x				x	x		x		**R**	gs		x			x		x		x	
8			x			x		x		x				x	x		x		x		R	**ls**	ls		x		x		x	
9			x			x		x		x				x	x		x		x			x		ls	**ls**	R	**ls**	R	x	
10			x			x		x		x				x	x		x		x			x			x		x		**R**	gs

R: reference condition; x: condition used in experiment; ns: test condition which is not significant at *α* = 0.01 in two-sided hypothesis test; ls: test condition which is significant at *α* = 0.01 in one-sided hypothesis test with alternative hypothesis true location shift is less than 0; gs: test condition which is significant at *α* = 0.01 in one-sided hypothesis test with alternative hypothesis true location shift is greater than 0; bold type: optimal condition as concluded from the experiment.

^a^300: single colony in 300 *µ*L sterile deionised water; 100: single colony in 100 *µ*L sterile deionised water; 50: single colony in 50 *µ*L sterile deionised water; IGM: InstaGene Matrix.

**Table 2:** Summary of the effect of optimisation on signal-to-noise ratios and on cost of the multiplex oligonucleotide ligation-PCR assay.

MOL-PCR step	Optimised parameter	Effect of optimisation	
		Signal-to-noise ratios	Cost of the assay
Preparatory work	DNA isolation	++	+

Multiplex oligonucleotide ligation	Probe concentration	++	+
	DNA volume	+	—
	*Taq* DNA ligase	+	++^a^
	Initial denaturation	+	—

PCR	Ligation product volume	—	—
	*Taq* DNA polymerase	+	++^b^
	Cycles	+	—

Hybridisation and analysis on MAGPIX	PCR product volume	+	—
	Microspheres per reaction	++	++^c^
	SAPE concentration	++	++^d^
	Reporter dye	+	+

++: major effect; +: minor effect; —: no effect; MOL-PCR: multiplex oligonucleotide ligation-PCR. ^a^Factor 2-3 for the cost of the *Taq* DNA ligase used; ^b^Factor 2 for the *Taq* DNA polymerase used; ^c^Factor 3.3 for the amount of microspheres per reaction used; ^d^Factor 2.5 for the amount of SAPE per reaction used.
