# Supplementary material for: Guidelines for Optimisation of a Multiplex Oligonucleotide Ligation-PCR for Characterisation of Microbial Pathogens in a Microsphere Suspension Array
Source: Biomed Res Int. 2015 Feb 3;2015:790170. doi: 10.1155/2015/790170 (PMC4332752; doi:10.1155/2015/790170)
Supplement: Supplementary file 1 — The supplemental figures show the boxplots of signal-to-noise ratios of additional parameters. The supplemental data sets include the MFI values that were used to calculate the signal-to-noise ratios as background information to explain some of the results. [file 790170.f1.pdf]

Supplementary figures

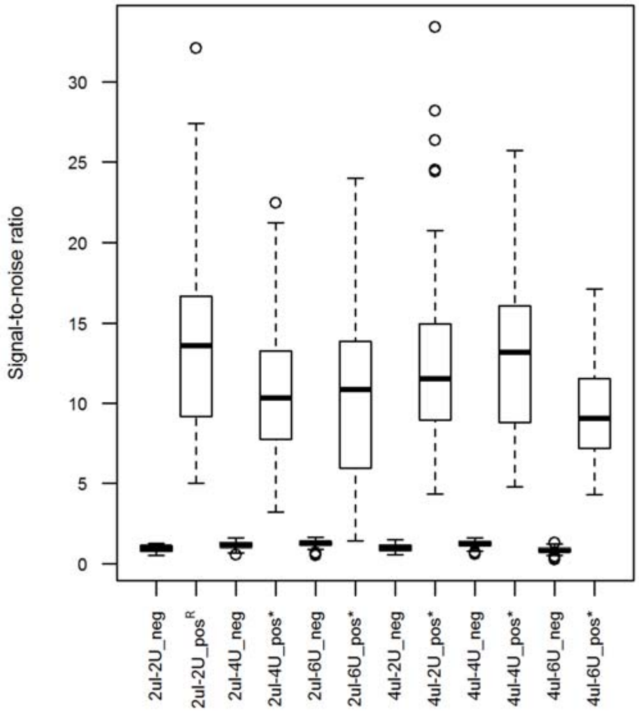

FIGURE S.1: Boxplot of signal-to-noise ratios for different combinations of DNA template volume and units of *Taq* DNA ligase in the ligation mix. Results of one of 2 independent tests are presented, *i.e.* results of 6 isolates x 6 conditions = 36 MOL-PCR assays (for each of the 6 conditions: n_{total}=120 with n_{neg}=64 and n_{pos}=56). 2ul: 2 µl of DNA template; 4 ul: 4 µl of DNA template; 2U: 2 units of *Taq* DNA ligase; 4U: 4 units of *Taq* DNA ligase; 6U: 6 units of *Taq* DNA ligase; neg: expected negative results; pos: expected positive results; R: reference condition; *: statistically significant difference with the reference condition.

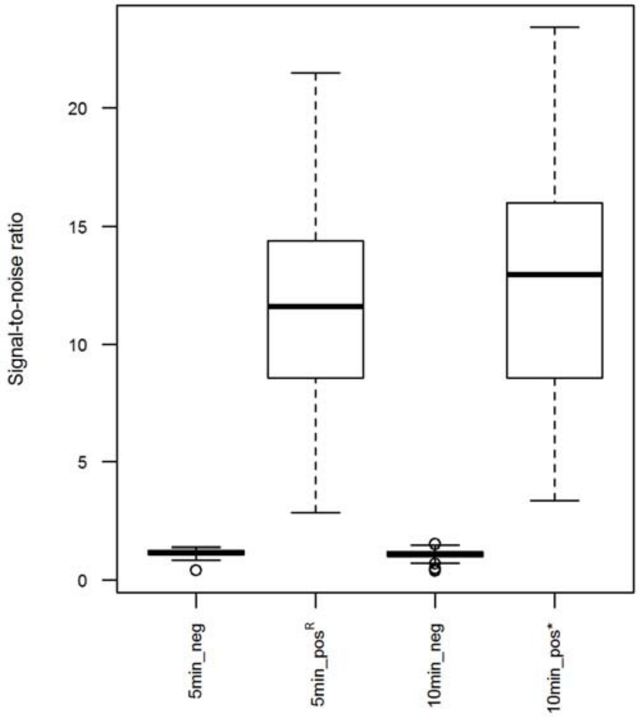

FIGURE S.2: Boxplot of signal-to-noise ratios for different initial denaturation times in the multiplex oligonucleotide ligation. Results of one of 2 independent tests are presented, *i.e.* results of 6 isolates x 2 conditions = 12 MOL-PCR assays (for each of the 2 conditions: n_{total}=120 with n_{neg}=64 and n_{pos}=56). neg: expected negative results; pos: expected positive results; R: reference condition; *: statistically significant difference with the reference condition.

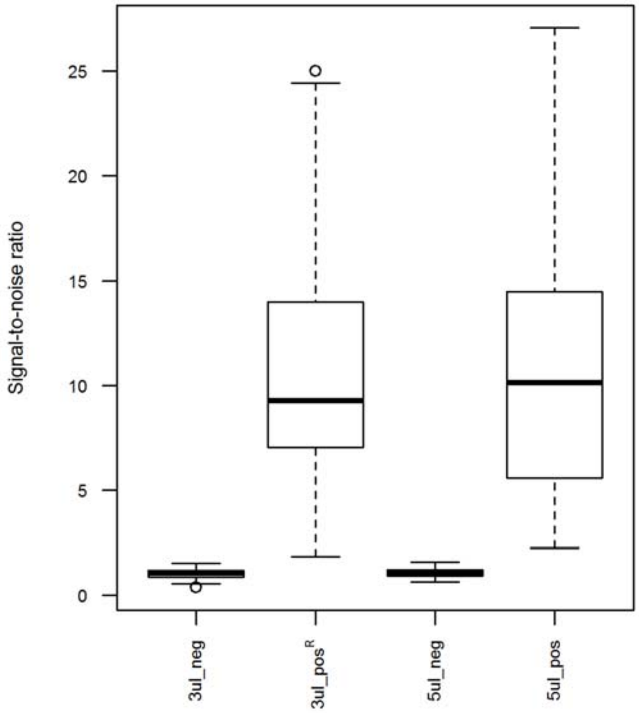

FIGURE S.3: Boxplot of signal-to-noise ratios for a volume of 3 µl and 5 µl of ligation product added to the PCR mix. Results of one of 2 independent tests are presented, *i.e.* results of 6 isolates x 2 conditions = 12 MOL-PCR assays (for each of the 2 conditions: n_{total}=120 with n_{neg}=64 and n_{pos}=56). 3ul: 3 µl of ligation product; 5ul: 5 µl of ligation product; neg: expected negative results; pos: expected positive results; R: reference condition.

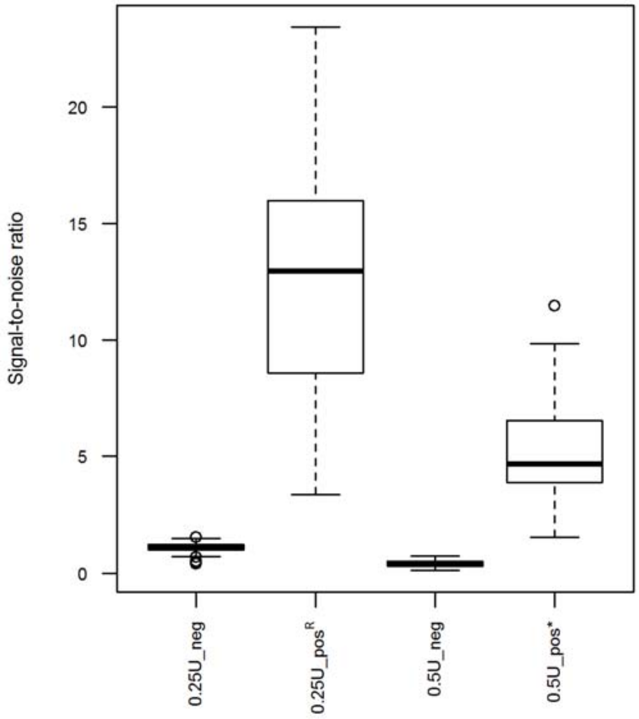

FIGURE S.4: Boxplot of signal-to-noise ratios for 0.25 units and 0.5 units of DNA polymerase the PCR mix. Results of one of 2 independent tests are presented, *i.e.* results of 6 isolates x 2 conditions = 12 MOL-PCR assays (for each of the 2 conditions: n_{total}=120 with n_{neg}=64 and n_{pos}=56). 0.25U: 0.25 units of DNA polymerase; 0.5U: 0.5 units of DNA polymerase; neg: expected negative results; pos: expected positive results; R: reference condition; *: statistically significant difference with the reference condition.

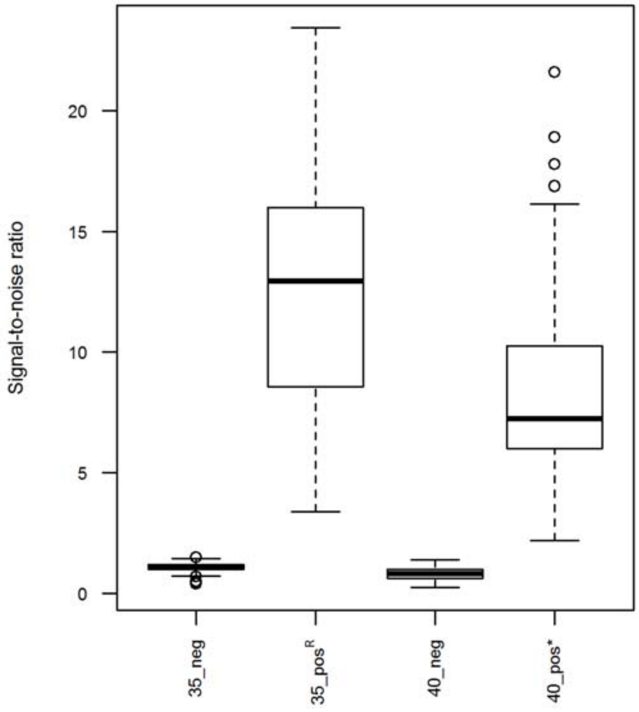

FIGURE S.5: Boxplot of signal-to-noise ratios for a PCR reaction with 35 and 40 cycles. Results of one of 2 independent tests are presented, *i.e.* results of 6 isolates x 2 conditions = 12 MOL-PCR assays (for each of the 2 conditions: n_{total}=120 with n_{neg}=64 and n_{pos}=56). neg: expected negative results; pos: expected positive results; R: reference condition; *: statistically significant difference with the reference condition.

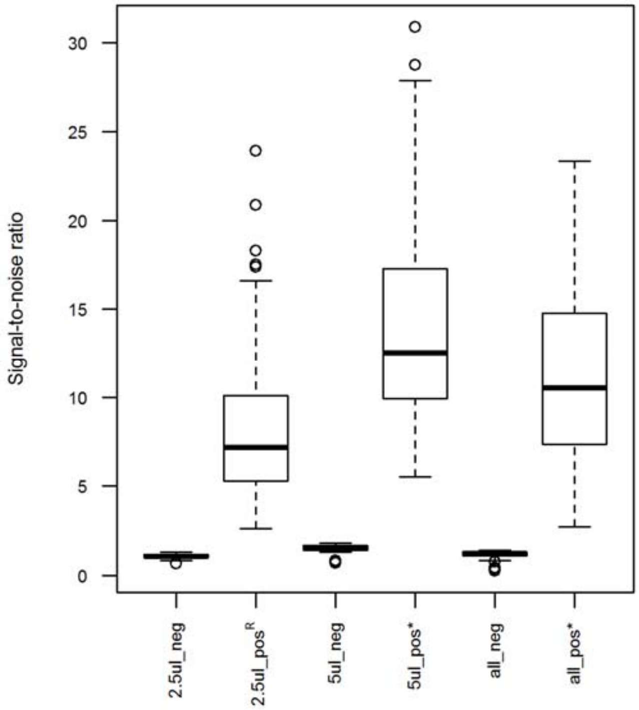

FIGURE S.6: Boxplot of signal-to-noise ratios for hybridisation of different volumes of PCR product to the MagPlex-TAG^{TM} microspheres. Results of one of 2 independent tests are presented, *i.e.* results of 6 isolates x 3 conditions = 18 MOL-PCR assays (for each of the 3 conditions: n_{total}=120 with n_{neg}=64 and n_{pos}=56). 2.5ul: 2.5 µl of the PCR product was added to the hybridisation reaction; 5ul: 5 µl of the PCR product was added to the hybridisation reaction; all: all (theoretically 10 µl) of the PCR product was added to the hybridisation reaction; neg: expected negative results; pos: expected positive results; R: reference condition; *: statistically significant difference with the reference condition.

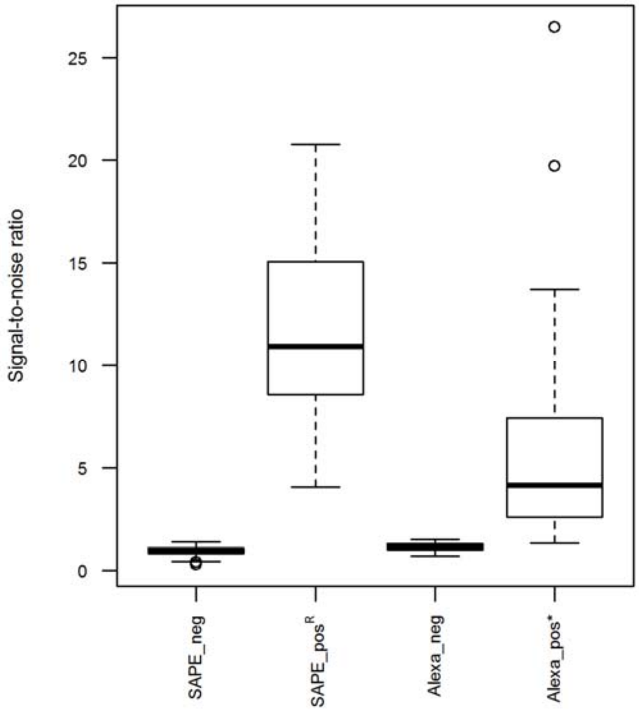

FIGURE S.7: Boxplot of signal-to-noise ratios for streptavidin-R-phycoerythrin (SAPE) and Alexa®532 as fluorescent reporters. Results of one of 2 independent tests are presented, *i.e.* results of 6 isolates x 2 conditions = 12 MOL-PCR assays (for each of the 2 conditions: n_{total}=120 with n_{neg}=64 and n_{pos}=56). SAPE: streptavidin-R-phycoerythrin as fluorescent reporter; Alexa: Alexa®532 as fluorescent reporter; neg: expected negative results; pos: expected positive results; R: reference condition; *: statistically significant difference with the reference condition.

Supplementary data sets

Data set S.1: Median fluoresence intensity (MFI) of expected positive results for different methods of DNA isolation (data set related to Figure 2)

Result	300ul_pos	50ul_pos	IGM_pos
1	427	884	745
2	297	509.5	894
3	748	742.5	887
4	592	932	1088
5	486	610	896
6	1184.5	510	194
7	891.5	1870	1587
8	625	1216	1885
9	1508	1575.5	1797
10	1284	1901.5	2099
11	944	1294	1811
12	2367	1130.5	672
13	146	332	296
14	343	466	394
15	170	274.5	332
16	326	815	634
17	330	1066	1027
18	757	904	829
19	340	533	832
20	183.5	405	383
21	215	334.5	456
22	415	997	818
23	906	1214	1021
24	501	684.5	922
25	459	941	780
26	529	709	1036
27	180	291	728.5
28	425	471	624
29	329	631	907
30	360.5	1174.5	1197
31	844.5	994	909
32	149.5	359	277
33	174	579	558.5
34	400.5	486.5	386
35	190	288	313
36	230	512.5	943.5
37	630	775	914
38	503	1058	1199.5
39	221	662	637
40	202	390	1010
41	174	383	321
42	186	301.5	420
43	372	1259	975
44	413.5	854.5	1475.5
45	1549	1150	579
46	167	596	400
47	184	348	691
48	527	959.5	974.5
49	156	400	373
50	290	728	649
51	302	480.5	856
52	1080.5	1643	1688
53	1184	1473.5	1818
54	1017	1592	1562
55	967	1309	1775
56	1828	1268	939
Mininum	146	274.5	194
Maximum	2367	1901.5	2099
Average	575.2	826.2	900.3

Data set S.2: Median fluoresence intensity (MFI) of the no-template-control (NTC) for different probe concentrations in the ligation mix (data set related to Figure 3)

Marker	2nM_NTC	1nM_NTC
Internal positive control_1	87.5	192
internal positive control_2	95	419
SAL-10	80	149
SAL-11	92	217
SAL-16	105	343
SAL-23	95.5	162
SAL-25	78	183
SAL-29	93.5	167
SAL-33	271	465
SAL-45	106	357.5
SAL-49	79	140
SAL-50	101.5	200
SAL-51	102	175
SAL-53	131	189
SAL-55	84	128
SAL-67	103	375
SAL-68	130	182
SAL-69	143	221
SAL-70	107	289.5
SAL-74	108	307
Mininum	78	128
Maximum	271	465
Average	109.6	243.1

Data set S.3: Median fluoresence intensity (MFI) of expected positive results for different probe concentrations in the ligation mix (data set related to Figure 3)

Result	2nM_pos	5nM_pos
1	977	659
2	245.5	595
3	1061	670
4	1060	702
5	956.5	640
6	1013.5	682
7	1819	1680
8	276.5	1624.5
9	1838	1742
10	1973.5	1791
11	1655.5	1670
12	1798	1799
13	745	447
14	1206	854.5
15	685	432
16	862.5	712
17	432.5	1150
18	1275.5	1105
19	801	732
20	587	388.5
21	526.5	330
22	931	874
23	1461	1366
24	871	820
25	1287	1244.5
26	1180	1142
27	110	424
28	749	484
29	1336.5	510.5
30	442	1454
31	1388	1446
32	798	344.5
33	526	766
34	1271	655
35	659.5	268
36	128.5	793
37	682.5	825
38	1325.5	950.5
39	1038	502
40	991	434
41	920	527
42	801	471
43	920	1214
44	892.5	1245
45	1770.5	1300
46	365.5	508
47	339	496
48	1310	1488
49	901	370.5
50	1411.5	1497
51	1315	1518
52	1701	1817
53	1555	1893
54	1738.5	1488
55	1602.5	1495.5
56	2022	1563.5
Mininum	110	268
Maximum	2022	1893
Average	1045.3	975

Data set S.4: Median fluoresence intensity (MFI) of the no-template-control (NTC) for 2 µl of DNA template volume with different numbers of *Taq* DNA ligase units in the ligation mix (data set related to Figure S.1)

Marker	2ul-2U_NTC	2ul-4U_NTC	2ul-6U_NTC
Internal positive control_1	67.5	90	93
internal positive control_2	73.5	96	104
SAL-10	71	82	87
SAL-11	77	91	87.5
SAL-16	81	101	88.5
SAL-23	78	107	85
SAL-25	70	94	88.5
SAL-29	74	90	82
SAL-33	113.5	111.5	121
SAL-45	88.5	95	96
SAL-49	75	84	87.5
SAL-50	75	86	90
SAL-51	73.5	81	83
SAL-53	66	75	83
SAL-55	63	86	73
SAL-67	70	80.5	84.5
SAL-68	99	130	168
SAL-69	82.5	91	99
SAL-70	67	75.5	84
SAL-74	70	90	99
Mininum	63	75	73
Maximum	113.5	130	168
Average	76.8	91.8	94.2

Data set S.5: Median fluoresence intensity (MFI) of expected positive results for different volumes of DNA template with 2 units of *Taq* DNA ligase in the ligation mix (data set related to Figure S.1)

Result	2ul-2U_pos	4ul-2U_pos
1	1020	1053
2	1058.5	1029
3	980.5	915
4	1416	1238.5
5	1100	893
6	1090	1082.5
7	1758	1784
8	1871	1809
9	1841	1668
10	2361	2160
11	1880	1625
12	2015	1995.5
13	631.5	687
14	1015	980.5
15	758.5	644
16	829.5	874
17	1208.5	1229
18	1208	1159
19	956	792
20	515	607.5
21	679	506
22	989	1017
23	1449	1370.5
24	1074	897
25	1036	1056
26	1185	887
27	488	469.5
28	555	497
29	899.5	628
30	1408.5	1443
31	1331	1353
32	636	725
33	1186	1208
34	1170	1069
35	791	631
36	739.5	732
37	704.5	657
38	1232	1022
39	497	436.5
40	628	413
41	449.5	479
42	551	402
43	778	748
44	872	680
45	994	1035.5
46	314	318
47	428	273.5
48	1129	1147
49	853	953.5
50	1014.5	938
51	1150	944
52	1630.5	1604.5
53	1702.5	1474
54	1376	1328
55	1475	1168
56	1604.5	1614
Mininum	314	273.5
Maximum	2361	2160
Average	1080.6	1006.3

Data set S.6: Median fluoresence intensity (MFI) of the no-template-control (NTC) and of expected positive results for different initial denaturation times in the multiplex oligonucleotide ligation (data set related to Figure S.2)

Marker	5min_NTC	10min_NTC
Internal positive control_1	92.5	77
internal positive control_2	107	94
SAL-10	91	77.5
SAL-11	96.5	81
SAL-16	127.5	125
SAL-23	90	79.5
SAL-25	89	75
SAL-29	96	74
SAL-33	104	98
SAL-45	116	113
SAL-49	84	63
SAL-50	94	79.5
SAL-51	89	77
SAL-53	88	63.5
SAL-55	82	63
SAL-67	76	73
SAL-68	231	197
SAL-69	85	78
SAL-70	79	72
SAL-74	105.5	87
Mininum	76	63
Maximum	231	197
Average	101.2	87.4

Result	5min_pos	10min_pos
1	797	767
2	984	907
3	881	762
4	1087	1057
5	947.5	892
6	991.5	968
7	1886	1883.5
8	2106	2046.5
9	2113.5	1905
10	2300	2201.5
11	2097	2051
12	2174	2159
13	566	531.5
14	983	896.5
15	772.5	672
16	911.5	895
17	1338	1309
18	1285	1157.5
19	1079	1069
20	712	688
21	927	841
22	1029.5	963
23	1560	1405
24	1241.5	1203
25	975	957
26	1118.5	1133
27	657	554
28	503	365.5
29	828	755.5
30	1500	1393
31	1442	1288.5
32	841	738
33	1620.5	1515
34	1449	1184.5
35	1096	952.5
36	1072	952
37	821.5	694
38	1227	1207
39	570	439
40	724	614
41	446.5	401
42	534	555.5
43	1102	915.5
44	1264	1173
45	1452	1310
46	444	390
47	549.5	532
48	1128	1155
49	655	662
50	968.5	823
51	1174	1100
52	1400	1368
53	1494	1465.5
54	1348	1240
55	1461.5	1344
56	1674	1535
Mininum	444	365.5
Maximum	2300	2201.5
Average	1148.4	1070.4

Data set S.7: Median fluoresence intensity (MFI) of the no-template-control (NTC) and of expected positive results for 0.25 units and 0.5 units of DNA polymerase in the PCR mix (data set related to Figure S.4)

Marker	0.25U_NTC	0.5U_NTC
Internal positive control_1	92.5	322
internal positive control_2	107	593.5
SAL-10	91	277
SAL-11	96.5	425
SAL-16	127.5	994
SAL-23	90	380
SAL-25	89	166
SAL-29	96	325
SAL-33	104	403
SAL-45	116	756.5
SAL-49	84	170.5
SAL-50	94	395
SAL-51	89	232
SAL-53	88	316
SAL-55	82	175
SAL-67	76	220
SAL-68	231	285
SAL-69	85	151.5
SAL-70	79	204
SAL-74	105.5	494
Mininum	76	151.5
Maximum	231	994
Average	101.2	364.3

Result	0.25U_pos	0.5U_pos
1	797	1277.5
2	984	1479
3	881	1153
4	1087	1555
5	947.5	1395
6	991.5	1538.5
7	1886	2736.5
8	2106	2999
9	2113.5	2707
10	2300	2949
11	2097	2972
12	2174	3065
13	566	1017
14	983	1368
15	772.5	1208.5
16	911.5	1563
17	1338	2014
18	1285	1761
19	1079	1745
20	712	1499
21	927	1648
22	1029.5	1892.5
23	1560	2141.5
24	1241.5	2046
25	975	1463
26	1118.5	1623
27	657	1023
28	503	732
29	828	1359
30	1500	1742
31	1442	1512
32	841	1684
33	1620.5	2588
34	1449	2102.5
35	1096	1887
36	1072	1627
37	821.5	1449
38	1227	1953
39	570	1300
40	724	1527.5
41	446.5	895
42	534	1067
43	1102	2206
44	1264	2412
45	1452	2634
46	444	1060.5
47	549.5	1341
48	1128	1763
49	655	1495
50	968.5	1296
51	1174	1491.5
52	1400	1880.5
53	1494	1968
54	1348	2161
55	1461.5	2244.5
56	1674	2499
Mininum	444	732
Maximum	2300	3065
Average	1148.4	1780.6

Data set S.8: Median fluoresence intensity (MFI) of the no-template-control (NTC) and of expected positive results for a PCR reaction with 35 and 40 cycles (data set related to Figure S.5)

Marker	35_NTC	40_NTC
Internal positive control_1	92.5	163.5
internal positive control_2	107	431
SAL-10	91	103.5
SAL-11	96.5	192
SAL-16	127.5	470
SAL-23	90	169
SAL-25	89	99.5
SAL-29	96	135.5
SAL-33	104	200.5
SAL-45	116	389
SAL-49	84	112
SAL-50	94	151.5
SAL-51	89	148
SAL-53	88	117
SAL-55	82	99.5
SAL-67	76	116
SAL-68	231	119
SAL-69	85	135
SAL-70	79	86
SAL-74	105.5	210.5
Mininum	76	86
Maximum	231	470
Average	101.2	182.4

Result	35_pos	40_pos
1	797	1017
2	984	1076.5
3	881	1060
4	1087	1295
5	947.5	1249
6	991.5	1323
7	1886	2275
8	2106	2308
9	2113.5	2426
10	2300	2602
11	2097	2553
12	2174	2662
13	566	647
14	983	1056.5
15	772.5	921.5
16	911.5	1162
17	1338	1393
18	1285	1411.5
19	1079	1383
20	712	1017
21	927	1352
22	1029.5	1274.5
23	1560	1807
24	1241.5	1624.5
25	975	1371
26	1118.5	1679.5
27	657	817
28	503	682
29	828	1276
30	1500	1414
31	1442	1378
32	841	1070
33	1620.5	1799
34	1449	1590
35	1096	1379
36	1072	1210.5
37	821.5	1155
38	1227	1542
39	570	635
40	724	899
41	446.5	665
42	534	918
43	1102	1464
44	1264	1890
45	1452	2079.5
46	444	681
47	549.5	1055
48	1128	1415
49	655	818
50	968.5	1111
51	1174	1394
52	1400	1624
53	1494	1857
54	1348	1636
55	1461.5	1880
56	1674	2082
Mininum	444	635
Maximum	2300	2662
Average	1148.4	1417.2

Data set S.9: Median fluoresence intensity (MFI) of expected positive results for different combinations of amount of microspheres in the hybridisation reaction and streptavidin-R-phycoerythrin concentration in the reporter mix (data set related to Figure 4)

Result	2500-10ug_pos	375-4ug_pos	750-4ug_pos	2500-4ug_pos	375-10ug_pos	750-10ug_pos
1	697	1143	1091	490	1534	1397
2	656	1173.5	1150	447	1679	1380
3	637	1121	1061.5	476	1471.5	1204.5
4	731	1563	1369.5	845	2055	1571
5	606	1116.5	1098	476.5	1626.5	1269
6	444	1224	1104	341.5	1676	1324
7	1421	2146	2196	1070	2871	2717
8	1411	2317	2244.5	935	3031	2680
9	1400.5	2145	2192	1089	2731	2494
10	1547	2479.5	2626.5	1720.5	3319	2991.5
11	1353	2150.5	2203	1074	2870	2548
12	918	2248.5	2281.5	852	3015	2691
13	445	627.5	662	240	925	873.5
14	593	1040	996	389	1444	1170.5
15	435	739.5	721.5	251	1130	837
16	760	1112.5	1119	524.5	1483.5	1419.5
17	1064	1465	1394	726.5	1905	1726.5
18	1021	1431	1316	735.5	1817	1620.5
19	714.5	1158	1165	532	1662	1354
20	493	796	906	299	1169	1103.5
21	434	797	915	278	1332	952
22	785	1448.5	1329	552	1801	1639
23	1138	1730	1656	834.5	2225	1907
24	755	1331	1356.5	546	1864	1512
25	680	1131	1131	463	1530	1475.5
26	600.5	1088	1137.5	437.5	1523.5	1275
27	393	691	628.5	198	927.5	777
28	421	544	593	252.5	771	735
29	479	1025.5	1057	353.5	1286	1028
30	1198.5	1305.5	1454	785	1813.5	1842
31	1173	1358	1428	821	1673	1655.5
32	571	909	992.5	387	1250	1202.5
33	965	1566	1595.5	665	1916.5	1658
34	822.5	1341	1399.5	613	1698	1518
35	523.5	918	992.5	350	1349	1037
36	580	1234	1163	350	1509.5	1314.5
37	588	970	968	376	1247.5	1142
38	745	1722	1579	685	2052	1706
39	523.5	1005	699	310	1166	936
40	448	632	679	282	989	803
41	378	693	608.5	215.5	928	802
42	369	550.5	598	210	1030.5	689
43	819	1973	1594	662	2382.5	1947
44	657	1426.5	1556.5	566	2063.5	1615.5
45	498	1606	1472	453	2113.5	1686
46	416	908	737	246	1329.5	1013.5
47	361	649	750	234	1129.5	831
48	757	1549	1391	485	1866	1707
49	547	762	878.5	327	983	1037.5
50	815	1464	1286	536	1792.5	1608.5
51	779	1297	1310	559	1791	1461
52	982	1921	1687.5	658	2418	2157.5
53	971.5	1938.5	1723	700.5	2322.5	2020
54	924	1673	1549	660.5	2187	2029
55	779	1433	1538	616	2154	1748
56	685.5	1713	1683	597.5	2398	2037
Mininum	361	544	593	198	771	689
Maximum	1547	2479.5	2626.5	1720.5	3319	2991.5
Average	748.4	1312.5	1285.9	549.6	1754.1	1515.7

Data set S.10: Median fluoresence intensity (MFI) of the no-template-control (NTC) for different combinations of amount of microspheres in the hybridisation reaction and streptavidin-R-phycoerythrin concentration in the reporter mix (data set related to Figure 4)

Marker	2500-10ug_pos	375-4ug_pos	750-4ug_pos	2500-4ug_pos	375-10ug_pos	750-10ug_pos
Internal positive control_1	259.5	86	78	84	193	218
internal positive control_2	311	77	75	76	192	212
SAL-10	318	81	70	91.5	191	233
SAL-11	294	86	76	91	177	247.5
SAL-16	299	81.5	92	84	201	230
SAL-23	263.5	82	77.5	78	184.5	233.5
SAL-25	314	71.5	69.5	95	184	242
SAL-29	298	75.5	68	85	187	216
SAL-33	300.5	119	113	93	233.5	273
SAL-45	287	82	65	85	192	250
SAL-49	297	81	70	85	175	225
SAL-50	316	77.5	76	100	192.5	234
SAL-51	302	97.5	66.5	90	190	221
SAL-53	280.5	88	67	76	200	202.5
SAL-55	275.5	85	61	83	179	210
SAL-67	300	80.5	64	84	151.5	222
SAL-68	293	76	82	96	157	243
SAL-69	293	117	81	89.5	206	226
SAL-70	290.5	72	67	81	178	196
SAL-74	331	95.5	77	95	190	269.5
Mininum	259.5	71.5	61	76	151.5	196
Maximum	331	119	113	100	233.5	273
Average	296.2	85.6	74.8	87.1	187.7	230.2
